# Disease burden and adverse pregnancy outcomes due to cardiovascular conditions complicating pregnancy in Sri Lanka: a protocol

**DOI:** 10.12688/f1000research.52539.1

**Published:** 2021-10-11

**Authors:** Ayesh Hettiarachchi, Niroshan Lokunarangoda, Thilini Agampodi, Suneth Agampodi

**Affiliations:** 1 Department of Community Medicine, Faculty of Medicine and Allied Sciences, Rajarata University of Sri Lanka, Saliyapura, Anuradhapura, 5008, Sri Lanka; 2 Department of Medicine, Faculty of Medicine and Allied Sciences, Rajarata University of Sri Lanka, Saliyapura, Anuradhapura, 5008, Sri Lanka

**Keywords:** maternal, heart disease, pregnancy, cardiovascular, Sri Lanka, Anuradhapura

## Abstract

**Background**

Cardiovascular diseases (CVD) are the commonest indirect medical cause of maternal deaths worldwide, both in high-income and low and middle-income countries. To minimize the effects of CVD in pregnancy, proper risk assessment and appropriate referral is required. In Sri Lanka, cardiovascular disease complicating pregnancy is a significant cause of maternal mortality, second only to postpartum hemorrhage. Screening for CVD in pregnancy in Sri Lanka is limited to a routine clinical assessment. Evidence-based guidelines are yet to be developed, and this deficit may have resulted in a substantial underestimation of the CVD burden. This study aims to determine the burden of CVD in early pregnancy and develop a risk prediction model to be used in field pregnancy clinics in Sri Lanka to reduce CVD effects in pregnancy.

**Methods**

A prospective cohort study was carried out in the Anuradhapura district, Sri Lanka. Following registration to the antenatal care, pregnant women fulfilling the eligibility criteria were invited to attend a special clinic at their relevant Medical Officer of Health (MOH) area. Risk assessment was done through history and a clinical examination, and suspected/probable cases were referred for an echocardiogram by a consultant cardiologist. All the recruited participants in the first trimester were prospectively followed up and screened again between 24–28 weeks of the period of amenorrhoea (POA). Antenatal ward admissions with CVD complicating pregnancy will be extracted, and a telephone interview will be carried out between 6–12 weeks after the expected delivery date to cover postpartum morbidities.

**Discussion**

This proposed study will be the largest of its kind carried out in the local setting. The study's findings will be beneficial for policymakers to develop guidelines to reduce maternal cardiovascular disease morbidities and mortalities in Sri Lanka.

## Introduction

In 2017, an estimated 295,000 (uncertainty interval 279,000–340,000) women died due to pregnancy-related complications, with 86% of these deaths occurring in sub-Saharan Africa and south-east Asia ^1^. The sustainable development goal target 3.1, reducing the global maternal mortality ratio (MMR) to less than 70 per 100,000 live births United Nations: Transforming our world, seems still too far away with an estimated MMR of 211 per 100,000 live births currently. Despite global interventions, obstetric hemorrhage still accounts for 27·1% (19·9–36·2) of all maternal deaths, followed by hypertension (14·0%, 11·1–17·4), sepsis (10·7%, 5·9–18·6) and abortions (7·9%, 4·7–13·2) ^2^. While the direct causes are still contributing to most deaths, 27.5% are due to indirect causes, and the proportion is increasing. Despite this increasing trend, there is little focus by the leading international maternal health non-governmental organizations and UN organizations on these causes ^3^.

Cardiovascular diseases (CVD) are the commonest indirect medical cause of maternal deaths worldwide, both in high-income countries (HIC) and low and middle-income countries (LMIC) ^2^. Although CVD affects only 1–4% of pregnant women, cardiomyopathy is reported as a leading cause of indirect maternal death ^4^. The general hemodynamic changes during pregnancy with 40–45% increase of blood volume, up to 50% increase in cardiac output, 15–30% increase in heart rate together with changes in vascular resistance, renal blood flow, red cell volume, and structural changes ^5^ put the system into a state of stress, which can greatly increase the risk of CVD related mortality and morbidity during pregnancy. To minimize the effects of CVD in pregnancy, proper risk assessment and appropriate referral are required ^6^. The use of a risk index is always preferred for predicting maternal cardiac risk ^7, 8^. Several risk assessment models have been suggested for this purpose and are being used in many HICs. These include the CARPEG score by the Cardiac Disease in Pregnancy Study ^8, 9^, the Zahara model ^10^, and the World Health Organization (WHO) risk classification ^11^.

In Sri Lanka, cardiovascular disease complicating pregnancy is a major cause of maternal mortality, second only to postpartum hemorrhage ^12, 13^. National Maternal Mortality Reviews 2016 outcome dissemination ^12^ shows 120 maternal deaths in 2016, and of those, 24 (20%) were related to cardiovascular disease. These included, complicated congenital heart condition (n=5), rheumatic valvular heart disease (n=3), myocarditis (n=2), cardiomyopathy (n=1), HELLP syndrome (n=3), eclampsia (n=4), and other hypertensive disorders (n=2). In the year 2017, maternal deaths due to cardiovascular diseases were 20 out of 127 total maternal deaths, and it was only second to dengue hemorrhagic fever in that year ^14^.

Screening for CVD in pregnancy in Sri Lanka is limited to a routine clinical assessment. Evidence-based guidelines are yet to be developed for the screening of cardiovascular disease in pregnancy. This lack of proper screening may lead to a substantial underestimation of the CVD burden. Although it is the second leading cause of maternal deaths, locally available data on cardiovascular conditions complicating pregnancy are limited ^13, 15^, probably due to late diagnosis and underestimation.

## Objectives

The present study's main objective is to determine the burden of CVD in early pregnancy and determine the incidence of CVD during pregnancy. Findings of this study will be used to develop a risk prediction model to be used in field pregnancy clinics in Sri Lanka to reduce the effects of CVD in pregnancy.

## Methods

This study was conducted as a part of large ongoing cohort study in Anuradhapura district, Sri Lanka, Rajarata Pregnancy Cohort (RaPCo). Details of the study setting, study population, sampling procedure, and the study sample of the RaPCo study are published elsewhere ^16^. The specific protocol for cardiac disease complicating pregnancy is described here. 

### Study design

This is a prospective cohort study developed to determine the prevalence and the incidence of common CVD conditions in a cohort of pregnant females in the Anuradhapura district.

### Study setting

The present study was carried out in the Anuradhapura district, the largest Sri Lanka district. According to 2017 Department of Census and Statistics data ^17^, it covers 7,179 km ^2^ with a total population of 917,748. The total fertility rate of the district is 2.4 births per woman, one of the highest values in Sri Lanka ^18^.

**Figure 1.**
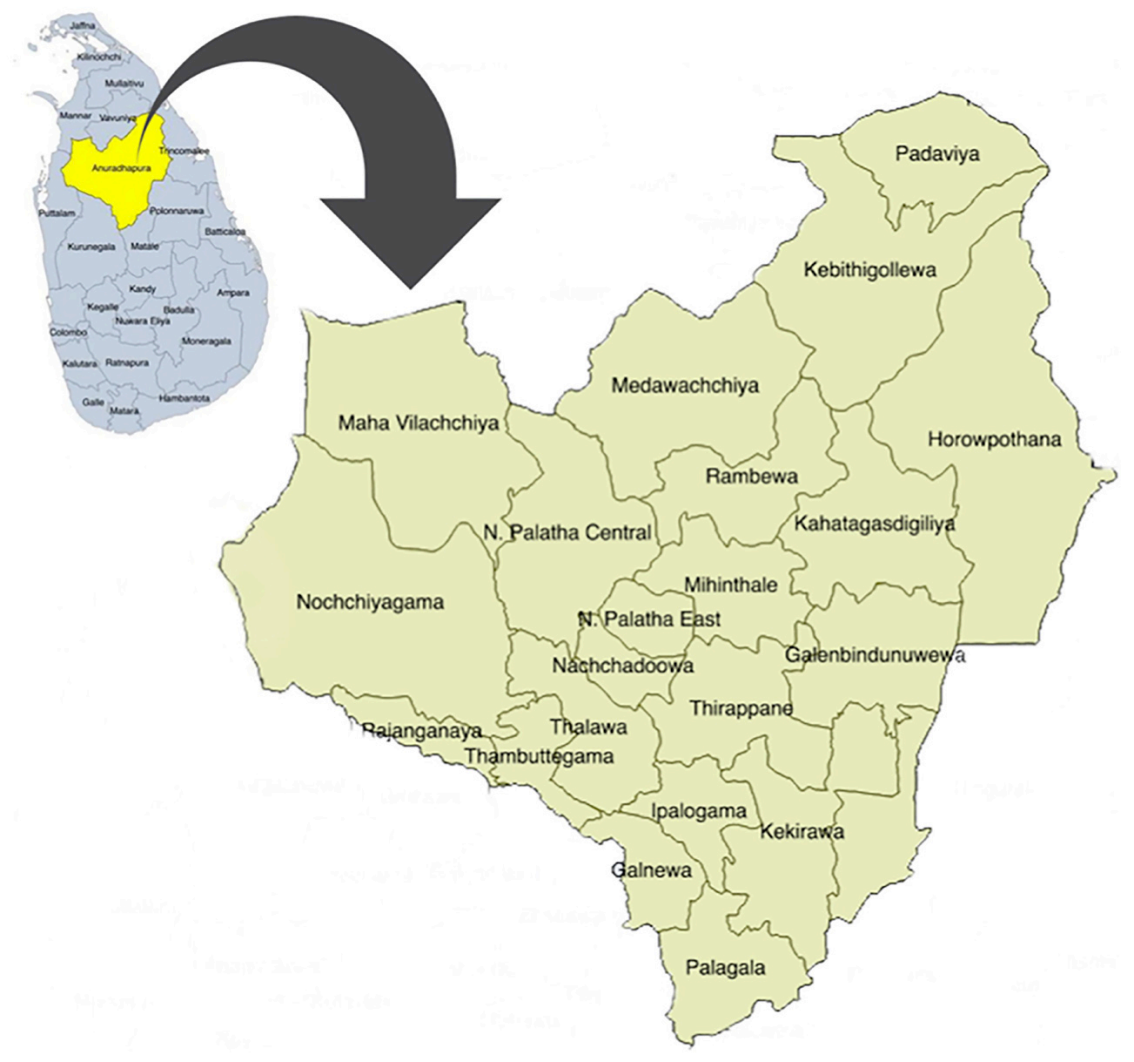
Spatial distribution of Medical Officer of Health (MOH) areas in Anuradhapura district, Sri Lanka. This figure shows the 22 MOH areas in the district. It has been reproduced with permission from Agampodi TC *et al*. The Rajarata Pregnancy Cohort (RaPCo): study protocol. BMC Pregnancy Childbirth [Internet]. 2020 Jun 26 [cited 2020 Nov 30];20(1):374. Available from: https://bmcpregnancychildbirth.biomedcentral.com/articles/10.1186/s12884-020-03056-x

In Sri Lanka, the field maternal and child health services are mainly provided by the public health administrative units named "Medical Officer of Health" (MOH) areas. In each MOH area, a Medical Officer of Health (MOH) is in charge and leads a team of ground-level health care workers, including public health midwives (PHM), public health nursing sisters (PNS), and public health inspectors. In Anuradhapura, this service for pregnant women is provided through 22 MOHs, and currently 17 PNS and more than 250 PHM are working in the area. Spatial distribution of those MOH areas is displayed in Figure 1.

Curative services are provided through 56 hospitals/primary care units, including one teaching hospital (tertiary care unit) and three base hospitals (secondary care units). Almost all deliveries (99.5%) take place in government hospitals ^18^. The study was conducted in all 22 MOH areas covering all the antenatal clinics.

### Study population

All pregnant females in Anuradhapura district.

### Study sample

All pregnant females visiting antenatal clinics in the Anuradhapura district from July to September 2019.

### Inclusion criteria

Pregnant women:

1. Registered by field midwives and visiting field antenatal clinics in the Anuradhapura district.

2. Period of amenorrhoea (POA) less than 12 weeks by the time of recruitment.

### Exclusion criteria

1. Pregnant women planning to leave the study area for childbirth or after childbirth.

2. Pregnant women who are not registered at field antenatal clinics. 

### Sample size

**Figure 2.**
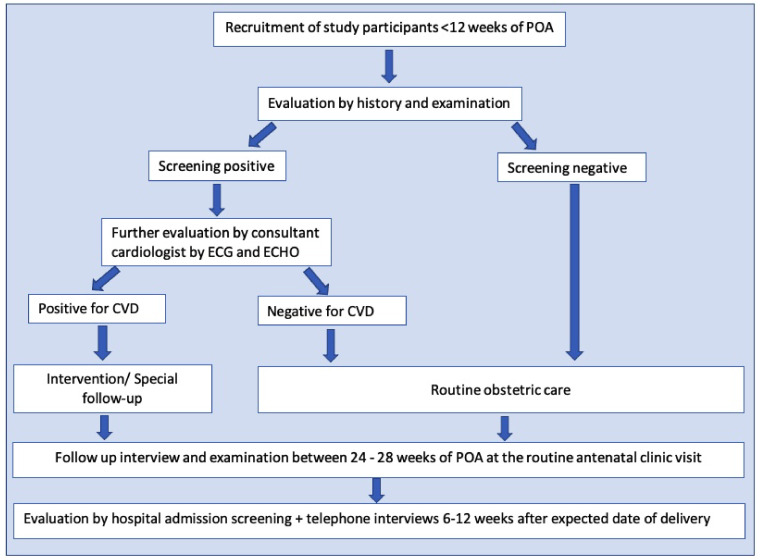
Flow chart of the study. The flow chart shows the developed methodology to achieve the study objectives. Following the registration to the antenatal care, pregnant women fulfilling the eligibility criteria were invited to attend a special clinic in their Medical Officer of Health area. Risk assessment was done through a specific interviewer-administered questionnaire, including clinical features of present cardiovascular diseases and a clinical examination. Based on these data and cardiac screening, suspected/probable cases with cardiovascular conditions were referred for an echocardiogram and consultant cardiologist's opinion. All the recruited participants in the first trimester were followed up between 24–28 weeks of the period of amenorrhoea at the routine antenatal clinic visit. They were screened again using a separate short interviewer-administered questionnaire (20) and were subjected to general and cardiac examination, referrals were done using a checklist (20). All antenatal ward admissions with cardiac disease complicating pregnancy and all intensive care unit admissions were also screened during the study period. A telephone interview was conducted during the antenatal period and between 6–12 weeks after the expected delivery date to cover all antenatal and postpartum morbidities.

All the pregnant females registered at field antenatal clinics in Anuradhapura within three months from the beginning of the study were screened ( Figure 2). The usual number of pregnant mothers registered in each quarter is around 4000. However, 15–20% are registered after a POA of 10 weeks. Around 3000 pregnant women were recruited as the sample size. We hypothesized that the incidence of CVD complicating pregnancy among those who were without risk factors would be around 5% compared to 2% among those who did not have risk factors. With a conservative estimate of unexposed to exposed ratio 5:1, power of 80%- and two-sided confidence at 95% a sample of at least 1529 pregnant women (Kelsey formula) were required for the study. With around 15% early pregnancy miscarriages and 10% expected attrition, a minimum of 1911 pregnant women were required, and the number was well within the number recruited. Based on the actual rate of CVD, we will calculate the power of the study to detect the expected outcomes retrospectively.

### Study implementation procedure and recruitment of study participants

Following registration to the antenatal care, pregnant women fulfilling the eligibility criteria were invited to attend a special clinic in their relevant MOH area. This invitation was given by way of a leaflet provided to the antenatal women through the PHM. At the special clinic, potential participants were recruited by the data collectors who visit these special antenatal clinics (ANC) in the field. Data collectors visited each field antenatal clinic in all 22 MOH areas on the pre-planned special clinic day. Eligible pregnant mothers were educated about the research purpose and written informed consent was obtained before recruitment. Participants were informed that they might need to visit the Teaching Hospital, Anuradhapura, if the study suggested an echocardiogram. All the other data collection and follow-up was done at the routine antenatal clinics. Recruitment was carried out until the data collection period was over.

### Cardiovascular risk assessment

We carried out the risk assessment through a specific interviewer-administered questionnaire, including clinical features of present cardiovascular diseases ^19^ and a clinical examination. The examination included a baseline physical examination and referral checklist, including a specific set of variables/signs, each defined previously, to improve the validity and reliability of data ^19^. Measurement of blood pressure and CVS examination auscultation was done using standard protocols given to all medical officers doing the clinical assessment ^19^. Specially trained medical graduates carried out data collection to use the above equipment using standard guidelines, which was issued in print for each procedure ^19^.

According to the baseline data, every recruited mother was classified according to the New York Heart Association (NYHA) classification. Based on that data and cardiac examination screening, suspected/probable cases with cardiovascular conditions were referred to the Professorial Unit, Teaching Hospital, Anuradhapura, for an echocardiogram and consultant cardiologist's opinion ( Figure 2).

We developed this checklist with less stringent criteria to improve the sensitivity with low threshold values to develop a more refined screening checklist based on the results of the study. The development of the screening checklist was done after a literature review and expert opinions. We incorporated this checklist into the examination sheet to minimize the duplication of work.

### Referral procedure

As the objective was to determine the prevalence of common cardiac conditions in the first trimester of the pregnant female, the referral method was developed to have a low threshold. If any of the above signs or symptoms were positive, the participants were referred to the consultant cardiologist at the Professorial Unit, Teaching Hospital, Anuradhapura.

### Performing echocardiogram

All screening positive patients were subject to a two-dimensional (2D) echocardiogram and an electrocardiogram (ECG) to evaluate cardiac electrical activity and rhythm. The ECG was taken using the Zonecare, iMAC 300 Three channel ECG machine, and the 2D echocardiogram using a Philips EPIQ 7. 

### Patient follow up

All the recruited participants in the first trimester were followed up between 24–28 weeks of POA at the routine antenatal clinic visit. A separate short interviewer-administered questionnaire was used ^19^. We also carried out a general and cardiac examination, and referrals were done using a checklist ^19^.

Participants were followed up through the routine system, and all maternal morbidities and hospital admissions recorded through a specific hospital surveillance system. All antenatal ward admissions with cardiac disease complicating pregnancy were extracted and cross-matched with the initial cohort to determine whether there were conditions missed through the screening. This was done in two major hospitals with a consultant obstetrician where all pregnant mothers from Anuradhapura districts are transferred. To determine the near misses and severe complications, all intensive care unit admissions were also screened during the study period. We matched all pregnant admissions with the original cohort to identify any cardiac disease complicating pregnancy.

A telephone interview was conducted with all study participants to document all antenatal morbidities in addition to the hospital admission screening. The interviews were carried out between 6–12 weeks after the expected delivery date to cover postpartum morbidities. Records relating to those who reported any hospital admissions were extracted from relevant hospitals. This approach was proposed to minimize unnecessary in-person contacts during the COVID-19 pandemic.

### Bias

To reduce the bias in sample selection and recruitment all the mothers who have registered at the field antenatal clinics were given an equal opportunity to participate in the study. All the participants who fulfilled the inclusion and exclusion criteria were recruited to the study.

When it comes to the examination to reduce the observer bias, digital blood pressure monitors were used with the same method in every clinic. Two readings are taken from both arms with a five-minute gap and the lower value recorded as the blood pressure. If a high blood pressure value is detected (SBP> 135mmHg or DBP >85mmHg), a third reading is taken after a rest period of five minutes. The same procedure is carried out in subsequent blood pressure measurements of the participants.

To reduce the bias when referring participants for further assessment, referrals were initiated based solely on screening positivity at the initial assessment.

### Outcomes

The main focus of the study is to describe the actual disease burden of cardiovascular disease in pregnancy in Sri Lanka (prevalence and incidence). With a proper assessment of disease burden, we expect to propose strategies to be included in the national program.

A field model will be developed using the findings for early detection of cardiovascular conditions during pregnancy at field level. With the appropriate inputs from the stakeholder, this model will be used to propose the required changes in strategies related to control and prevention of CVD complicating pregnancy in Sri Lanka.

### Data management

All the data collection will be done using the electronic data collection method using a unique barcode and serial number. The database will be automatically formulated through this data collection platform. Access to the databases will be under restricted access. Access will be granted to principal investigators only. Two-dimensional echocardiogram reports will also be formulated via database management software, and a copy of the report will be provided to the participant. This database will also be accessible by the researchers only, and the data will be stored under the unique serial number.

### Data analysis

Data will be analysed using the Statistical Package for Social Sciences ( SPSS) version 26 (IBM SPSS Statistics, RRID:SCR_019096). An open-access alternative that can provide an equivalent function is the R stats package (R Project for Statistical Computing, RRID:SCR_001905).Point prevalence of cardiovascular diseases complicating pregnancy will be calculated with 95% confidence intervals (CI). The prevalence of each specific condition will also be presented as proportions. The cumulative incidence of heart disease incidence will be estimated, and the 95% CI will be calculated using a Poisson distribution. An analysis of factors associated with cardiovascular disease complicating pregnancy will be done, and relative risks will be calculated. A risk prediction model will be developed to identify the predictive factors of cardiac disease complicating pregnancy. This model will be used to develop a screening checklist for the public health system.

### Ethical considerations

All study procedures were done following the Helsinki declaration. Research was carried out only after the informed written consent of the participant for participation and after agreeing to publication of data collected in an anonymized format. Participants have been provided with the opportunity to withdraw from the study at any time. Participant identification data will be kept under lock and key to protect confidentiality. All identified conditions will be managed and treated by the consultant cardiologists with appropriate investigations and referral without an additional cost to the participant. Ethical clearance for the "Rajarata Pregnancy Cohort" has been obtained from the Ethics Review Committee, Faculty of the Medicine & Allied Sciences, Rajarata University of Sri Lanka under the approval number ERC/2019/07.

### Confidentiality

All the recruited participants have been provided with a serial number and a unique bar code. All the material and data belonging to a participant will be labelled with the above serial number and bar code. All the consent forms will be stored separately under lock and key at the Maternal and Child Health Research Unit, Faculty of Medicine and Allied Sciences, Rajarata University of Sri Lanka. The participant has also received an identity card with basic details and a barcode.

### Dissemination of findings

Findings will be published as research articles, abstracts, and presentations. Generated knowledge will be communicated with the local health care authorities, and nationally important findings will be published as policy briefs.

## Discussion

This study on cardiovascular disease in pregnancy is the largest study conducted of its kind in Sri Lanka. The patterns and associations identified with regard to cardiovascular disease during pregnancy and its distributions among the population will give an idea about the true picture of the disease in the LMICs. As this study focuses on both prevalence and incidence of cardiovascular disease during pregnancy it will provide a wider understanding about risks and predictive symptoms and signs of CVD during pregnancy in Sri Lankans. This will enable development of a model for early CVD risk identification among pregnant females. Also, the findings will have an impact on policy change to go beyond routine screening methods.

This project proposal was developed in June 2019. Recruitment of participants was done from July to September 2019. The first round of referral and cardiovascular assessment and part of the second trimester assessment is already completed. However, the follow-up was interrupted due to the COVID-19 pandemic and lockdown since March 2020. Based on the country’s current situation, delivery and outcome data collection has been initiated through alternative methods. Telephone interviews and data extraction from labor rooms are also in progress. Records relating to those who reported any hospital admissions during the telephone interviews will be extracted from the relevant hospitals.

## Data availability

### Extended data

Open Science Framework: Generating Evidence for Ending Preventable Maternal Deaths (GEEPMED) in Sri Lanka. Extended data for ‘Disease burden and adverse pregnancy outcomes due to cardiovascular conditions complicating pregnancy in Sri Lanka’, ‘Tools’. https://doi.org/10.17605/OSF.IO/RTDCG ^19^

This project contains the following extended data:

1. Baseline questionnaire

2. Baseline physical examination and referral checklist sheet

3. Definition of selected signs and symptoms

4. Guide for blood pressure measurement

5. Guide for CVS examination

6. Follow-up questionnaire

7. Follow-up examination and referral checklist sheet

Data are available under the terms of the Creative Commons Attribution 4.0 International license (CC-BY 4.0)
