# Supplementary material for: Disease burden of cardiovascular conditions complicating pregnancy in Sri Lanka: a protocol
Source: F1000Res. 2024 Feb 29;10:1028. Originally published 2021 Oct 11. [Version 4] doi: 10.12688/f1000research.52539.4 (PMC10948970; doi:10.12688/f1000research.52539.4)
Supplement: Peer review history

# Reviewer report: Reviewer response for version 4

**DOI:** 10.5256/f1000research.163166.r251735

**Published:** 2024-03-18

**Authors:** Andrea Sonaglioni

**Affiliations:** 1 MultiMedica IRCCS, Sesto San Giovanni MI, Italy

The Authors have exhaustively answered to my suggestions. I do not have any other suggestions.

Is the study design appropriate for the research question?

Yes

Is the rationale for, and objectives of, the study clearly described?

Yes

Are sufficient details of the methods provided to allow replication by others?

Yes

Are the datasets clearly presented in a useable and accessible format?

Yes

Reviewer Expertise:

Cardiology, Echocardiography

I confirm that I have read this submission and believe that I have an appropriate level of expertise to confirm that it is of an acceptable scientific standard.

# Reviewer report: Reviewer response for version 3

**DOI:** 10.5256/f1000research.122032.r226942

**Published:** 2024-02-21

**Authors:** Andrea Sonaglioni

**Affiliations:** 1 MultiMedica IRCCS, Sesto San Giovanni MI, Italy

The manuscript describes an elegant modality of cardiovascular risk assessment in pregnancy in Sri Lanka.

 The screening modality is mainly based on two-dimensional echocardiography and electrocardiography.

 I congratulate the Authors for their work.

 I have only one suggestion for the Authors.

 In the Introduction section, after the following sentence "Screening for CVD in pregnancy in Sri Lanka is limited to a routine clinical assessment. Given that evidence-based guidelines are yet to be developed for the screening of cardiovascular disease in pregnancy", I suggest the Authors to describe an innovative screening modality for detecting subclinical myocardial dysfunction, that is employed in Europe since one decade, i.e. the speckle tracking echocardiography. 

 The Authors could add a short paragraph for describing strain echocardiographic imaging in pregnancy, They could cite and discuss following references ref [1,2]

Is the study design appropriate for the research question?

Yes

Is the rationale for, and objectives of, the study clearly described?

Yes

Are sufficient details of the methods provided to allow replication by others?

Yes

Are the datasets clearly presented in a useable and accessible format?

Yes

Reviewer Expertise:

Cardiology, Echocardiography

I confirm that I have read this submission and believe that I have an appropriate level of expertise to confirm that it is of an acceptable scientific standard, however I have significant reservations, as outlined above.

## References

[1] : Cardiac maladaptation in obese pregnant women at term. *Ultrasound Obstet Gynecol* .2019;54(3) : 10.1002/uog.20170 344-349 10.1002/uog.20170 30381850

[2] : The impact of short-term hyperglycemia and obesity on biventricular and biatrial myocardial function assessed by speckle tracking echocardiography in a population of women with gestational diabetes mellitus. *Nutr Metab Cardiovasc Dis* .2022;32(2) : 10.1016/j.numecd.2021.10.011 456-468 10.1016/j.numecd.2021.10.011 34893411

# Reviewer report: Reviewer response for version 3

**DOI:** 10.5256/f1000research.122032.r127630

**Published:** 2022-03-16

**Authors:** Anish Keepanasseril

**Affiliations:** 1 Department of Obstetrics and Gynaecology, Jawaharlal Institute of Postgraduate Medical Education & Research, Puducherry, Tamil Nadu, India

Thank you for revising the manuscript based on the comments.

Is the study design appropriate for the research question?

Yes

Is the rationale for, and objectives of, the study clearly described?

No

Are sufficient details of the methods provided to allow replication by others?

Partly

Are the datasets clearly presented in a useable and accessible format?

Not applicable

Reviewer Expertise:

NA

I confirm that I have read this submission and believe that I have an appropriate level of expertise to confirm that it is of an acceptable scientific standard.

# Reviewer report: Reviewer response for version 2

**DOI:** 10.5256/f1000research.118272.r120418

**Published:** 2022-01-28

**Authors:** Anish Keepanasseril

**Affiliations:** 1 Department of Obstetrics and Gynaecology, Jawaharlal Institute of Postgraduate Medical Education & Research, Puducherry, Tamil Nadu, India

Thank you for revising the manuscript and the opportunity to review again.

 The following points are not clear again in the revision.

1. Please give under the subheading definitions of the outcome. How they will be identified, with references if they are already described elsewhere? This is required to avoid misclassification of the outcomes.
2. The aim is to develop a predictive model for the development of cardiovascular complications and some of the outcomes which you have described might have been already present but not yet identified. So they are not incidence of the outcomes described (as it is explained in the responses). So how will the factors (which might be prevalent before the pregnancy) be addressed in the model?
3. As it is given,k two models (probably) will be developed: one for the diagnostic algorithm and the other suggesting the adverse outcomes. Please separate both of them as they have different interpretations and usefulness for clinicians.
4. Please refer to the PROGRESS group website detailing the development of the prediction model and the analysis. You can revise the methods as well as the analysis based on them.
5. How do you intend to minimise the bias arising from lost to follow up?

Is the study design appropriate for the research question?

Yes

Is the rationale for, and objectives of, the study clearly described?

No

Are sufficient details of the methods provided to allow replication by others?

Partly

Are the datasets clearly presented in a useable and accessible format?

Not applicable

Reviewer Expertise:

NA

I confirm that I have read this submission and believe that I have an appropriate level of expertise to confirm that it is of an acceptable scientific standard, however I have significant reservations, as outlined above.

# Response

**Published:** 2022-03-02

**Authors:** Ayesh Hettiarachchi

**Affiliations:** Rajarata University of Sri Lanka, Sri Lanka

Many thanks for reviewing my article again. Please see the following clarifications for the points raised.

1. Please give under the subheading definitions of the outcome. How they will be identified, with references if they are already described elsewhere? This is required to avoid misclassification of the outcomes.
  - Thank you for the comment. Definitions are included. See the response below as well.
2. The aim is to develop a predictive model for the development of cardiovascular complications and some of the outcomes which you have described might have been already present but not yet identified. So they are not incidence of the outcomes described (as it is explained in the responses). So how will the factors (which might be prevalent before the pregnancy) be addressed in the model?
  - Thank you very much for the comments. The way we presented the “outcome” was slightly confusing as you pointed out. We are looking at the CVD related events (mentioned as incidence) leading to hospital admissions and adverse pregnancy outcomes (previously mentioned as pregnancy outcomes). Since the way it was presented was not clear (thank you for the question), have edited the title and relevant places. As you have pointed out, we will have data on previous conditions (prevalence) during the baseline assessment. However, the outcome we are interested in is what really predicts the CVD events in pregnancy (with or without knowledge on pre-existing CVDs).  
3. As it is given, two models (probably) will be developed: one for the diagnostic algorithm and the other suggesting the adverse outcomes. Please separate both of them as they have different interpretations and usefulness for clinicians.
  - Agree and added to the methods.
4. Please refer to the PROGRESS group website detailing the development of the prediction model and the analysis. You can revise the methods as well as the analysis based on them.
  - Thank again. We have included that we will adhere to the PROGRESS group suggestions given in developing, validating and evaluating the model. 
5. How do you intend to minimise the bias arising from lost to follow up?
  - Many thanks for pointing out this deficit. We have included a comprehensive, methodology to minimize the lost to follow up. I’ve added a paragraph under the subheading of Bias.

# Reviewer report: Reviewer response for version 1

**DOI:** 10.5256/f1000research.55834.r96617

**Published:** 2021-11-11

**Authors:** Anish Keepanasseril

**Affiliations:** 1 Department of Obstetrics and Gynaecology, Jawaharlal Institute of Postgraduate Medical Education & Research, Puducherry, Tamil Nadu, India

The study aims to assess the burden of CVD among women in the first trimester of pregnancy and to develop a risk prediction model to be used in Sri Lankan hospitals.

 Even though the study has already started according to the protocol, the following are my suggestions:

**Objective**

1. It needs to be more specific about the study. The incidence of CVD may be difficult to determine unless we have a pre pregnancy check up and have the details available. It may be better to use the prevalence, as it will include the cases which are there in pre pregnancy as well as those who develop during the pregnancy.
2. Kindly mention the outcome for which the predictive models are being developed. Is it to identify people with the CVS or for identifying those who are at risk of adverse outcomes. Risk models can stratify and you might need to consider a future study or a subset wherein this stratification is useful either by a temporal or an external validation of the model.

**Methodology**

1. The design of the study, inclusion and exclusion criteria are well presented and helpful for performing/guiding a study to be planned else where. However, further reading it was unclear about the study design as well as about the intention of adding matched controls which is  highlighted in point 3. If any secondary objective is planned please provide the details in the objectives section of the protocol.
2. It is unclear which are the diseases that are included as CVD. As heart disease, hypertension, stroke etc. could be taken as CVD. More clarity is required on the outcomes.
3. How and why was the matching performed under patient follow up? Did the matched controls also undergo the same procedure? How did you avoid the selection bias in choosing controls? Did you look at any nested case control design? If so all these need to be more clarified in the text. Being an observational study, this is the main area of interest to the reader.
4. Detail about how the model will be built needs to be included. 

Is the study design appropriate for the research question?

Yes

Is the rationale for, and objectives of, the study clearly described?

No

Are sufficient details of the methods provided to allow replication by others?

Partly

Are the datasets clearly presented in a useable and accessible format?

Not applicable

Reviewer Expertise:

Maternal medicine

I confirm that I have read this submission and believe that I have an appropriate level of expertise to state that I do not consider it to be of an acceptable scientific standard, for reasons outlined above.

# Response

**Published:** 2021-12-20

**Authors:** Ayesh Hettiarachchi

**Affiliations:** Rajarata University of Sri Lanka, Sri Lanka

Thank you reviewing this paper. Please find a point by point response for the comments below.

**Comment 1 -** The study aims to assess the burden of CVD among women in the first trimester of pregnancy and to develop a risk prediction model to be used in Sri Lankan hospitals.

**Response 1 -** To clarify the confusion, this study aims to develop a risk prediction model to be used in Sri Lanka field health care setup not in the Sri Lankan hospitals.

**Comment 2 - **It needs to be more specific about the study. The incidence of CVD may be difficult to determine unless we have a pre pregnancy check up and have the details available. It may be better to use the prevalence, as it will include the cases which are there in pre pregnancy as well as those who develop during the pregnancy.

**Response 2 - **Thank you for the comment. And I’m sorry for the confusion. This study recruit first trimester pregnant females and screen them for CVD conditions. As you mentioned, we determine the prevalence of CVD conditions during the first trimester pregnant females. This change was done in the first objective.

 In addition, we do follow up the same cohort of pregnant females at the second trimester of their pregnancy. Then at the post-partum period. This follow up section of the study is planned to determine the incidence of CVD among the same cohort of pregnant females. This part is clearly shown in the flow diagram provided and at the outcomes section. To avoid the confusion, I’ve changed the first sentence under the subheading “objectives”.

**Comment 3 -** Kindly mention the outcome for which the predictive models are being developed. Is it to identify people with the CVS or for identifying those who are at risk of adverse outcomes. Risk models can stratify and you might need to consider a future study or a subset wherein this stratification is useful either by a temporal or an external validation of the model.

**Response 3 -**Thank you for the comment. This predictive model is being developed to identify pregnant women with adverse pregnancy outcomes due to existing or an emerging CVD condition. The objective is edited as suggested.

**Comment 4 - **The design of the study, inclusion and exclusion criteria are well presented and helpful for performing/guiding a study to be planned else where. However, further reading it was unclear about the study design as well as about the intention of adding matched controls which is  highlighted in point 3. If any secondary objective is planned please provide the details in the objectives section of the protocol.

**Response 4 -** Thank you for the kind comment. There are no matched controls in this study. This is a cohort study where a selected group of individuals recruited, screening and then follow-up throughout the pregnancy and the post-partum period. It is unclear from where this confusion is arising. Probably the misunderstanding is about the sentence “All antenatal ward admissions with cardiac disease complicating pregnancy were extracted and cross-matched with the initial cohort”. This was not a matched control group. To make sure we are not missing data, we separately looked at hospital admissions during the period and confirmed whether there are patients from our cohort who are having CVD related complications, but not captured during the follow up. We have edited the sentence.

**Comment 5 - **It is unclear which are the diseases that are included as CVD. As heart disease, hypertension, stroke etc. could be taken as CVD. More clarity is required on the outcomes.

**Response 5 - **Thank you for the valuable comment. I have included a statement under the cardiovascular risk assessment section. All the acquired or congenital heart conditions and other related cardiovascular conditions including hypertension, heart failure and pulmonary hypertension and arrythmias considered as CVD related to this study. History Cerebrovascular events were taken during the data collection for the risk prediction of related CVD condition.   

**Comment 6 - **How and why was the matching performed under patient follow up? Did the matched controls also undergo the same procedure? How did you avoid the selection bias in choosing controls? Did you look at any nested case control design? If so all these need to be more clarified in the text. Being an observational study, this is the main area of interest to the reader.

**Response 6 -** Kindly see my reply under your comment number 1 of methodology part. 

**Comment 7 - **Detail about how the model will be built needs to be included. 

**Response 7 -** Thank you for the comment. Details included under the data analysis section.
